# Analysis of the Impact of Disaggregates Government Expenditure on Economic Growth in Iraq

**DOI:** 10.12688/f1000research.177997.2

**Published:** 2026-08-21

**Authors:** Abdullah Qasim Mohammed, Muhannad Khamis Abd

**Affiliations:** 1 College of Administration and Economics, University of Fallujah, Al-Fallujah, Al Anbar Governorate, Iraq

**Keywords:** Investment spending, Current spending, Inflation, Economic growth, Iraq

## Abstract

**Background:**

Iraq heavily depends on oil revenues, which expose it to global oil price fluctuations as well as structural crises. Therefore, in the 2004–2023 crisis period, Iraq experienced economic shocks that followed the change in global prices and internal disturbances that undermined the stability and an enabling environment for growth. The government spending in the last five years has increased; however, its contribution to economic growth has been limited due to poor targeting on productive investment-driven expenditure and high reliance on current spending in the budget.

**Methods:**

This study adopts a quantitative methodology using time series data for the period 2004–2023 in Iraq. The Autoregressive Distributed Lag (ARDL) model was used, which is employed to analyse the short- and long-term correlation between economic variables.

**Results:**

Finally, the econometric tests findings indicate a long-term cointegration correlation between government spending components, oil prices, and the inflation rate, and economic growth. There was a direct correlation between investment spending, education spending, and oil barrel price and economic growth, and an inverse correlation between current spending, health spending, and the inflation rate and economic growth.

**Conclusions:**

While the impact of the current spending factor is of low intensity, investment spending is an active factor in stimulating economic growth in Iraq. Therefore, it is important to reorient the fiscal regime to support investment fields with production tendency and introduction of reform policies that promote economic elasticity and weak dependence on oil money.

## Introduction

This analysis research is concerned with studying the structure and evolution of government spending in Iraq for the period (2004–2023), with the main goal of identifying how the nature of distribution between financial resources allocated to current and investment spending and measuring the differential impact of each of the two components on economic activity using the ARDL model that allows studying the correlation in the short- as well as long-term. Moreover, we aim to explore the extent to which external factors such as oil price shocks and global economic conditions can change the correlation between the two components and determine the future course of fiscal policy in Iraq. This research has several objectives. First, it seeks to better understand the dynamics of this correlation in Iraq’s economy, for example, this correlation has become particularly relevant for the recent years following the massive price shocks that hit the oil market and the significant structural disruptions that the economy underwent. Finally, the last goal of the research is encouraging policymakers to adopt more efficient and flexible fiscal policies that would help sustain the economic activity and shift away from excessive dependence on oil revenues by directing government spending toward more production sectors with a higher potential to significantly contribute to achieving the economy’s diversification and stability.

## Background

### First: concept and types of government spending

Government expenditures relate to a state, regional, or municipal government’s entire spending. The purpose of this mechanism is to ensure economic transformation rates and is one of the most significant elements of gross national product in nearly all states. ^7^ It can be seen as a sum of money from the state general budget or an agency’s budget catering to the public’s well-being. ^3^

1. Development Investment Expenditures

It consists of long-term expenditures to enhance infrastructure and assist economic growth, including developing roads, schools, hospitals, and constructing service networks. This will boost productivity or lower production costs by stimulating investment and generating job chances in the long term, which might help increase domestic product. ^6^

2. Recurrent Expenditures

This pertains to repetition operational overheads to guarantee the continuity of the work of state organizations, such as salaries overheads, maintenance, and public services. Such a way of administering expenditures guarantees the stability of the activity of state service providers but does not add new kinds of assets or directly raise efficiency. ^8^

3. Other Expenditures

Other expenditures are diversion from plan without any adequate planning or during emergency or unexpected state such as funding disaster response or exceptional projects. It is highly flexible and offers the mechanism to respond to crises. Nevertheless, its long-term developmental impact is limited unless directed within well-planned schemes. ^13^

### Second: The concept of economic growth and methods of measuring it

As a constant and non-temporary phenomenon globally, the concepts of economic growth have changed. It is one of the complex terms because it directly influences the national economy and national income indicators. This is supposed to be due to the movement of market forces. ^2^ Economic growth is the improvement of gross domestic product, which occurs because of the combination of the growth rate of the population and improved productivity of the individual. Thus, the growth of GDP represents the answer to the improvement of living standards and the modernization of the economic structure of society. ^11^ There are three ways to determine it:

1. Per capita average: One of the most important indicators of economic growth and development is the average per capita income. It is calculated by the growth of per capita output over a long period as limited, provided the growth rate of GDP exceeds the growth rate of the population. A growth in personal income leads to an increase in the standard of living, which in its turn is positively reflected on production and productivity, increase in national income, and improvement in the general welfare. This indicator was used primarily for more than five decades since the 1960s; it served as a fundamental instrument to identify developed countries from developing countries. ^5^ The real per capita income is calculated, as demonstrated in equation: ^9^

Realpercapita income rate=(Gross Domestic Product/Population)

2. Output method: The output method relies on considering only the final market magnitude of the gross domestic product, meaning that primary or intermediate goods cannot be included in the components of the domestic product, as they are already included in the final goods. Therefore, calculating them again leads to double counting. ^10^ GDP is calculated mathematically dependent on the following equation. ^12^

Gross Domestic Product=(Total Production Magnitude–Production Inputs Magnitude)

3. Gross National Income method: Economic growth refers to the continuous increase in gross national product over a certain period, with the necessity of distinguishing between the level of real national income, which reflects the economic capacity of the state, and the national income growth rate, which expresses the efficiency of the economic system in achieving change and growth. Moreover, a high annual growth rate shortens the time needed to reach higher levels of national income. ^4^

## Methods

Research Methodology: To achieve the research objective and test its hypothesis, the descriptive (analytical) approach within economic theories and the quantitative (econometric) approach dependent on the econometric method using the cointegration methodology were adopted, relying on the statistical software (Eviews 12).

Descriptive Analysis: Analyzes the reality of government expenditure components, oil prices, and inflation rates, in addition to their correlation with economic growth in Iraq during the period (2004–2023).

Econometric Analysis: Analyzes the correlation between government expenditure components, oil prices, and inflation rate and their impact on economic growth. We will use an econometric approach dependent on instrumental variable models. First, we will test for stationarity, then test for cointegration between the economic variables under study, and third, find predicts for long-run and short-run factors and the error correction term, and then conduct diagnostic tests for the model.

## Results

**Results of Descriptive Analysis**: Analysis of the correlation between government expenditure components, oil prices, and inflation and their impact on economic growth.

**Figure 1.**
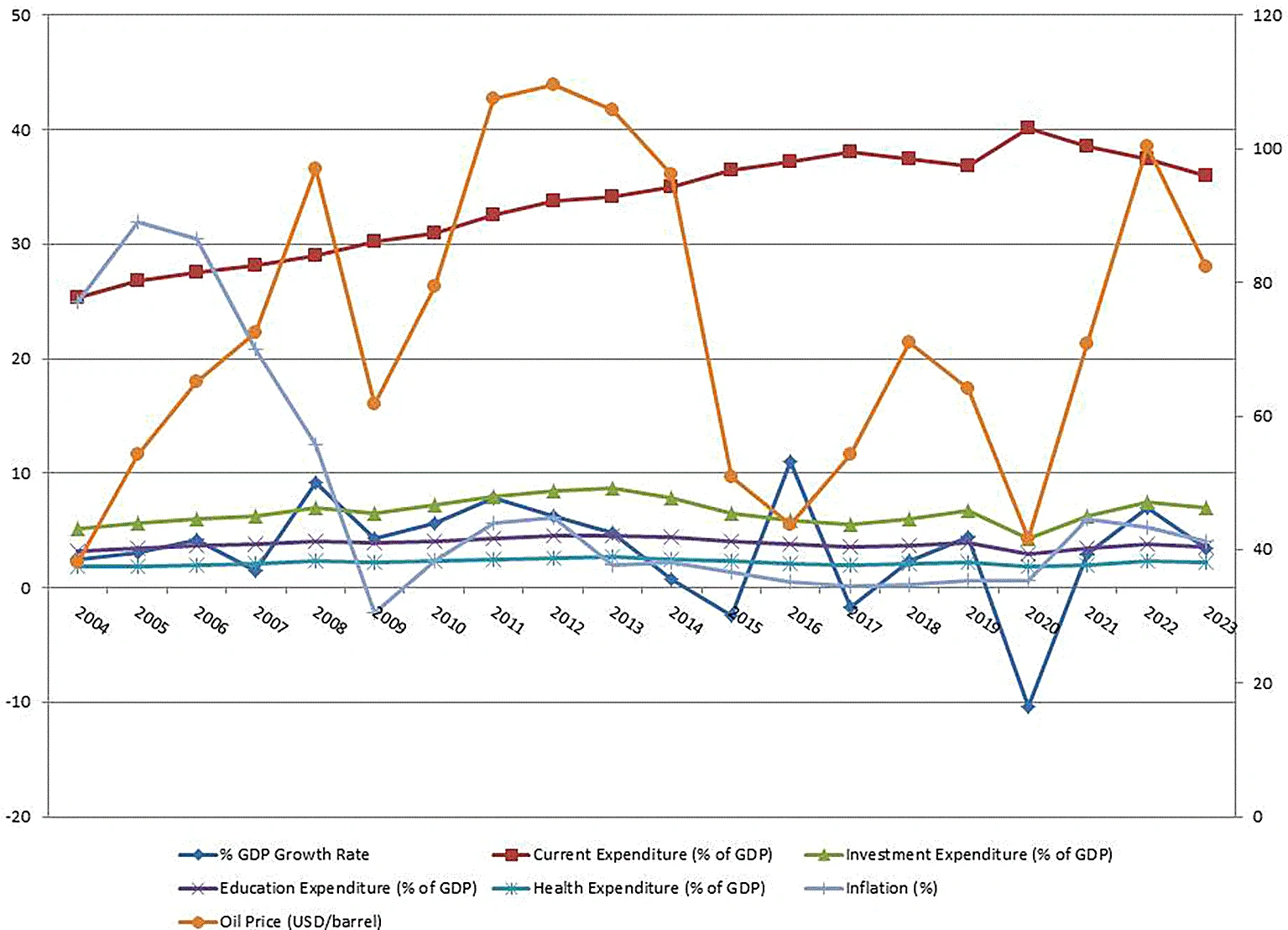
The correlation between government expenditure components, oil prices, and inflation, and their impact on the rate of change in GDP. Source: Authors’ calculations based on data available at Zenodo ( https://doi.org/10.5281/zenodo.19666496).

Figure 1 shows how government expenditure components, oil price, and inflation rate affect the dependent variable (annual GDP change rate). GDP growth and current expenditure as a proportion of GDP were inversely related. Spending increased from 25.3% in 2004 to 37.4% in 2022, while growth fluctuated between 2.5% and 7% and declined to 10.4% in 2020 with expenditure at 40.1%. This expenditure is going toward operational consumption rather than productive investment, which defies economic theory that ties well-planned government spending to growth. Investment expenditure as a proportion of GDP has a moderate positive association, rising to 6.2% in 2012 with 8.7% expenditure and 11% in 2016 with 5.9%. The 2020 crisis caused growth to decline to (−10.4%) despite expenditure at (6.8%), showing how sensitive this association is to economic and political settings. Despite the increase in health expenditure to 3.8% in 2016, GDP grew 11% due to increased oil prices, not health sector efficiency. Health expenditure grew to 3.9% in 2020 while GDP fell to 10.4% due to the oil crisis and COVID-19 pandemic. This suggests that health expenditure did not increase real productivity or economic growth, which contradicts the economic theory that human capital investment (such as health) boosts labor force productivity and growth. Education spending as a percent of GDP is correlated. Education expenditure surged to 5.2% in 2012 and 4.8% in 2016, respectively, boosting economic growth to 6.2% and 11%. In 2022, the GDP grew 7% and education spending about 4.5%). Dependent on economic theory, increasing educational spending increases human capital efficiency and productivity. Oil prices, on the other hand, directly affect growth, rising to (97 dollars/barrel) in 2008 and (107.5 dollars/barrel) in 2011, respectively, and falling to (41.7 dollars/barrel) in 2020, causing growth to contract to (−10.4%), reflecting the high dependence on oil revenues. GDP growth and inflation are inversely related. In 2020, inflation was 0.6% with a contraction of −10.4%, in 2008, it was 12.5% with growth of 9.2% due to the oil boom, and in 2018, it was 0.3% with moderate growth of 2.3%, supporting the theory that high inflation pressures GDP growth.

**Findings of the econometric analysis: Findings of measuring the correlation between government expenditure components, oil prices, inflation, and their impact on economic growth.**

1. **Defining the model variables and functional specification:**

Table 1 demonstrates the research variables used in the econometric model, their symbols, and their functional specification.

**Table 1.** Functional specification of the variables used in the econometric model and their symbols.

Independent variables	Variable name	Variable type
X1	Current expenditure as a percent of GDP	Independent Variables (Explanatory)
X2	Investment expenditure as a percent of GDP	
X3	Education expenditure as a percent of GDP	
X4	Health expenditure as a percent of GDP	
X5	Oil price (USD/barrel)	
X6	Inflation rate	
Y	Economic Growth (Annual change in GDP)	Response Variable (Dependent)

Source: Authors’ own work.

Accordingly, the model for the impact of disaggregating government expenditure components on economic growth considering price shocks and structural disturbances can be formulated dependent on the following functional form:

Y=f(X1,X2,X3,X4,X5,X6) (1)

2. **Testing the stationarity of time series for research variables:**

Regression models dependent on time series data rely in their predictions on the assumption that these series are characterized by stationarity. However, non-stationary series (i.e., those containing a unit root) often lead to spurious regression findings. The reason for this is the nature of time series data, which are often affected by a general trend resulting from common conditions that are reflected in all variables and make them move in the same direction, despite the absence of a real causal correlation between them. Therefore, it becomes necessary to test the stationarity property for all variables under study and determine the integration order of each variable before proceeding with the prediction. Table 2 demonstrates the findings of the time series stationarity test for research variables dependent on the Phillips-Perron (PP) test. It is observed that the calculated probability magnitudes (prob) were greater than (5%) at the original level I(0), which means that the time series are non-stationary and suffer from the presence of a unit root in all three cases (with constant, with constant and trend, and without them). Therefore, the null hypothesis, which states the existence of a unit root, is accepted. To address this problem, a unit root test was performed for the first difference of the time series data, and the probability magnitudes (prob) became less than (5%), which means that the time series for the research variables became stationary and integrated of the first order I(1), whether with an intercept, with an intercept and a general trend, or without them. Therefore, the alternative hypothesis, which states that the time series are free of a unit root, is accepted.

3. **Formulation of the Econometric Model and prediction Methodology.**

**Table 2.** Findings of the unit root test for stationarity dependent on the Phillips-Perron (PP) test.

At Level								
		Y	X1	X2	X3	X4	X5	X6
With Constant	t-Statistic	−4.7181	−2.2843	−2.3587	−2.1507	−2.0305	−2.5634	−1.7512
	Prob.	*0.0016*	*0.1864*	*0.1655*	*0.2289*	*0.2724*	*0.1175*	*0.3914*
	result	***	n0	n0	n0	n0	n0	n0
With Constant & Trend	t-Statistic	−5.2893	1.1088	−2.2572	−2.2988	−1.9507	−2.4162	−1.4078
	Prob.	*0.0024*	*0.9997*	*0.4347*	*0.4147*	*0.5895*	*0.3604*	*0.8246*
	result	***	n0	n0	n0	n0	n0	n0
Without Constant & Trend	t-Statistic	−3.2041	1.7374	0.2253	0.1274	0.3088	−0.0181	−1.9877
	Prob.	*0.0030*	*0.9754*	*0.7407*	*0.7111*	*0.7645*	*0.6641*	*0.0472*
	result	***	n0	n0	n0	n0	n0	**

At First Difference								
		d(Y)	d(X1)	d(X2)	d(X3)	d(X4)	d(X5)	d(X6)
With Constant	t-Statistic	−17.4804	−3.5899	−4.5209	−4.5638	−3.5197	−4.2199	−3.4002
	Prob.	*0.0000*	*0.0171*	*0.0026*	*0.0024*	*0.0197*	*0.0048*	*0.0250*
	result	***	**	***	***	**	***	**
With Constant & Trend	t-Statistic	−17.2234	−4.5872	−4.4241	−4.8475	−3.5647	−4.0764	−4.0221
	Prob.	*0.0001*	*0.0097*	*0.0132*	*0.0060*	*0.0624*	*0.0250*	*0.0276*
	result	***	***	**	***	*	**	**
Without Constant & Trend	t-Statistic	−18.9430	−3.2238	−4.6507	−4.7308	−3.6216	−4.4667	−3.2436
	Prob.	*0.0001*	*0.0029*	*0.0001*	*0.0001*	*0.0011*	*0.0002*	*0.0028*
	result	***	***	***	***	***	***	***

(*), (**), (***) indicate significance at the 10%, 5%, and 1% levels, respectively, dependent on Mackinnon’s critical magnitudes, and (no) indicates non-significance.

After conducting the stationarity test for the time series of the research variables, it became appropriate to use the modern cointegration methodology dependent on the (ARDL) model and to predict the equilibrium correlation in the short and long run and analyze it using the following linear form:

$$∆Y_{t}=c+B_{1}\;y_{t-1}+B_{2}\;X_{\text{1t}-1}+B_{3}\;X_{\text{2t}-1}+B_{4}\;X_{\text{3t}-1}+B_{5}\;X_{\text{4t}-1}+B_{6}\;X_{\text{5t}-1}+B_{7}\;X_{\text{6t}-1}+\sum_{i=1}^{ɋ}\lambda_{1}∆y_{t-i}+\sum_{i=1}^{ɋ1}\lambda_{2}∆x_{1t-i}+\sum_{i=1}^{ɋ2}\lambda_{3}∆x_{2t-i}+\ldots .+\sum_{i=1}^{ɋ6}\lambda_{7}∆x_{6t-i}+\varepsilon t$$ (2)

Where:

Yt: Economic growth (annual rate of change in GDP), X1: Current expenditure as a percent of GDP, X2: Investment expenditure as a percent of GDP, X3: Education expenditure as a percent of GDP, X4: Health expenditure as a percent of GDP, X5: Oil price (dollar/barrel), X6: Inflation rate, βi: Long-run correlation factors, λi: Short-run correlation factors, ∆: First differences of variable magnitudes, q: Optimal lag length, ε_t: Random error term.

Quarterly data (80 observations) for the period (2004–2023) were used, applying the standard program equation and the linear form of the (ARDL) model for prediction to obtain stable linear correlations where the predicts are interpreted as marginal propensities. The time series data for the model variables are publicly available in the Zenodo repository ( https://doi.org/10.5281/zenodo.19666496).

4. **Results of the Econometric Model prediction.**

a) Testing the Long-Run Equilibrium Correlation between Model Variables.

To test whether there is a long-run equilibrium correlation between the independent variables under study and the dependent variable, the F-statistic magnitude is calculated. If the calculated F-statistic magnitude is greater than the upper bound of the critical F-magnitude, we reject the null hypothesis (H0) and accept the alternative hypothesis (H1), which states that there is cointegration between the variables (existence of a long-run equilibrium correlation). Conversely, if the calculated F-statistic magnitude is less than its upper bound critical magnitude, we accept the null hypothesis (H0) and reject the alternative hypothesis (H1). Table 3 demonstrates the findings of the bounds test for the (ARDL) model.

**Table 3.** Findings of the cointegration test for the (ARDL) model dependent on the bounds test.

Test Statistic	Magnitude	K
F-statistic	6.649300	6
	Critical Magnitude Bounds	
Significance	I0 Bound	I1 Bound
10%	1.99	2.94
5%	2.27	3.28
2.50%	2.55	3.61
1%	2.88	3.99

Source: Created by the authors dependent on the outputs of the statistical program (Eviews 12).

Table 3 demonstrates the findings of the Bounds Test, where the calculated F-statistic magnitude reached (6.649300), which is higher than the critical upper bound F-magnitude of (3.99) at a significance level of (1%). This means rejecting the null hypothesis and accepting the alternative hypothesis, indicating a long-term equilibrium correlation moving from the set of independent variables towards the dependent variable (annual percent change in GDP). This confirms the validity of the research hypothesis, which necessitates predicting the long-term and short-term factors and the error correction term.

b) Findings on predicting short-term and long-term factors and the error correction term for the (ARDL) model:

After confirming the existence of a long-term equilibrium correlation (cointegration) between the independent variables and the dependent variable (annual percent change in GDP), it is necessary to predict the long-term factors, short-term factors, and the error correction term. Table 4 illustrates this.

**Table 4.** Findings of predicting long-term and short-term factors and the error correction term for the (ARDL) model.

Dependent variable: Y		Included observations: 76 after adjustments		
Method: ARDL		Maximum dependent lags: 4 (Automatic selection)		
Sample (adjusted): 2005Q1 2023Q4		Selected Model: ARDL(4, 3, 1, 4, 4, 1, 0)		
Fixed regressors: C		Model selection method: Akaike info criterion (AIC)		
Dynamic regressors (1 lag, automatic): X1 X2 X3 X4 X5 X6				
Variable	Coefficient	Std. Error	t-Statistic	Prob.
D(Y1(−1))	0.615369	0.092124	6.679806	0.0000
D(Y1(−2))	0.486898	0.109173	4.459879	0.0000
D(Y1(−3))	0.402322	0.114991	3.498722	0.0010
D(X1)	−2.305555	1.569311	−1.469151	0.1478
D(X1(−1))	1.726799	2.002318	0.862400	0.3924
D(X1(−2))	2.830632	1.594960	1.774735	0.0818
D(X2)	0.594229	3.411857	0.174166	0.8624
D(X3)	22.60350	11.40737	1.981482	0.0528
D(X3(−1))	−16.51389	8.425866	−1.959905	0.0554
D(X3(−2))	−9.712659	6.877688	−1.412198	0.1638
D(X3(−3))	−15.41467	4.422957	−4.389523	0.0001
D(X4)	−38.36932	8.034132	−4.775788	0.0000
D(X4(−1))	18.37400	7.461104	2.462639	0.0171
D(X4(−2))	17.11082	7.092031	2.412683	0.0194
D(X4(−3))	23.04442	6.466863	3.563462	0.0008
D(X5)	0.149584	0.036424	4.106756	0.0001
D(x6)	−19.41467	4.422957	−4.389523	0.0001
Coint Eq(−1)	−0.705803	0.090850	−7.768860	0.0000
Coint eq = Y1 - (−0.4621*X1 + 13.4485*X2 + 11.8181*X3–70.2208*X4–0.0576*X5–0.3471*X6 + 45.4628)				
Long Run Coefficients				
Variable	Coefficient	Std. Error	t-Statistic	Prob.
X1	−0.462076	0.122610	−3.768674	0.0004
X2	13.44848	2.423247	5.549775	0.0000
X3	11.81809	3.314126	3.565975	0.0008
X4	−70.22082	13.71947	−5.118333	0.0000
X5	−0.057642	0.031111	−1.852796	0.0696
X6	−0.347127	0.058133	−5.971206	0.0000
C	45.46277	8.913524	5.100426	0.0000
R-squared	0.946901	Mean dependent var		3.373684
Adjusted R-squared	0.923415	S.D. dependent var		4.775735
S.E. of regression	1.321635	Akaike info criterion		3.647706
Sum squared resid	90.82941	Schwarz criterion		4.383727
Log likelihood	−114.6128	Hannan-Quinn criter.		3.941855
F-statistic	40.31766	Durbin-Watson stat		1.873955
Prob(F-statistic)	0.000000			

Source: Created by the authors dependent on the outputs of the statistical program (Eviews 12).

This indicates the existence of a cointegration correlation between the independent variables and the dependent variable. This is confirmed by the error correction term (Coint Eq(−1)), which is (−0.705803), negative and significant at a significance level less than (1%). This means that the two basic conditions for this coefficient are met, implying that (0.70) of the short-term errors are automatically corrected over time to reach long-term equilibrium. In other words, the short-term imbalance can be adjusted in the long term, thus returning to an equilibrium state. This indicates that the adaptation in the econometric model used was relatively slow.

c) Evaluation of the statistical and econometric quality of the predicted model (diagnostic tests of the model):

• Evaluation of the statistical quality of the model:

Adjusted R ^2^: The magnitude of the adjusted coefficient of determination for the model (Adj R ^2^ = 0.923415) indicates that the explanatory variables, which represent the determinants of income distribution inequality under study, explain (92%) of the change occurring in the dependent variable (annual rate of change in GDP), and the remaining (8%) is due to other random factors.

t-test: The prediction findings indicate the significance of the predicted model factors for the independent variables and the constant term in both the long and short terms, as demonstrated in Table 4.

F-statistic magnitude: Which was (40.31766) with a probability magnitude (prob F = 0), indicates the significance of the predicted model as a whole in explaining changes in the dependent variable.

From the above, the model is free from potential statistical problems.

**Figure 2.**
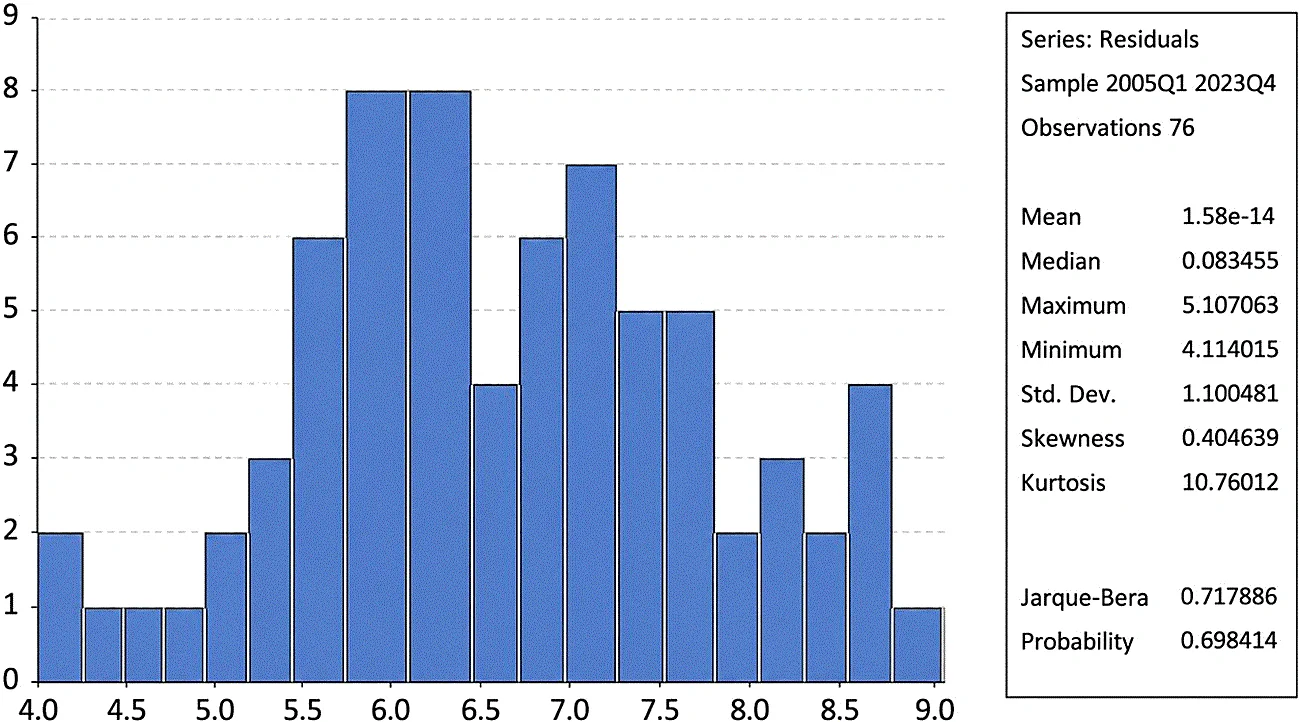
Normality test for the residuals of the predicted model. Source: Created by the authors dependent on the output of the statistical software (Eviews 12).

• Evaluation of the econometric quality of the model:

Normality test of residuals: It is clear from the magnitude of (JB = 0.717886) with a significance level higher than (5%), reaching (0.698414), that the error terms follow a normal distribution. This indicates the acceptance of the null hypothesis (H0) which states that the residuals follow a normal distribution, as demonstrated in Figure 2.

Autocorrelation test: Table 5 demonstrates the findings of the autocorrelation test for the predicted model. It is observed that the calculated F-statistic magnitude reached (0.1488) with a non-significant probability magnitude of (0.7026). Therefore, we accept the null hypothesis that there is no autocorrelation problem (H0:ρ = 0) in the model and reject the alternative hypothesis. This is supported by the Durbin-Watson statistic magnitude of (D.W = 2.0925).

**Table 5.** Findings of the Breusch-Godfrey Serial Correlation LM Test for the predicted ARDL model.

Breusch-Godfrey Serial Correlation LM Test			
F-statistic	0. 617886	Prob. F(2,50)	0.5431
Obs*R-squared	1.833069	Prob. Chi-Square(2)	0.3999

Source: Created by the authors dependent on the outputs of the statistical program (Eviews 12).

Heteroskedasticity Test: It is inferred from Table 6 that the predicted model does not suffer from the problem of heteroskedasticity, because the calculated F-statistic magnitude reached (1.148882) with a non-significant probability magnitude of (0.3306). This means accepting the null hypothesis, which states the constancy of the variance of the random error term of the predicted econometric model.

**Table 6.** Findings of the Heteroskedasticity Test for Random Error Terms.

Heteroskedasticity Test: ARCH			
F-statistic	1.148882	Prob. F(23,52)	0.3306
Obs*R-squared	25.60745	Prob. Chi-Square(23)	0.3197

Source: Created by the authors dependent on the output of the statistical program (Eviews 12).

Test of the functional form’s suitability for the model: Table 7 demonstrates the findings of the test for the suitability of the functional form for the model under study. The calculated F-statistic magnitude was (0.481320) with a probability magnitude of (0.4910), while the t-statistic magnitude was (0.693772) with a probability magnitude of (0.4910). Both probability magnitudes were higher than the significance level (5%), which means accepting the null hypothesis stating the correctness of the functional form specification used in the predicted econometric model.

**Table 7.** Findings of the Ramsey-RESET test for the suitability of the functional form for the (ARDL) model.

Ramsey-RESET Test			
Test	Magnitude	DF	Probability
t-statistic	0.693772	51	0.4910
F-statistic	0.481320	(1, 51)	0.4910

Source: Created by the authors dependent on the output of the statistical program (Eviews 12).

Structural Stability Test for Model Coefficients: Conducting a structural stability test for the short-term and long-term coefficients of the ARDL model requires ensuring that the data is free from any structural changes. This is done by using one of two tests: either the Cumulative Sum of Recursive Residuals (CUSUM) test or the Cumulative Sum of Squares of Recursive Residuals (CUSUM SQ) test, as demonstrated in the following figure:

It is clear from the figure above that the graphical line for the Cumulative Sum of Recursive Residuals (CUSUM) test falls within the critical limits (upper and lower limits) at a significance level of (5%), which means that the predicted coefficients for the Unrestricted Error Correction Model (UECM) used are structurally stable over the research period.

It is inferred from the findings of the diagnostic tests above the reliability of the predicts and findings obtained from the model, which indicates the quality of the predicted model and its freedom from measurement and statistical problems, and thus its findings can be relied upon and interpreted in a manner consistent with the economic reality of Iraq.

## Discussion

The following is inferred from the findings of the (ARDL) model prediction for the impact of government expenditure components on economic growth in the short and long run, as demonstrated in Table 4 above:

1. The current expenditure indicator as a percent of GDP suggests (X1) that the relationship between current expenditure as a percent of GDP and economic growth as annual rate of change in GDP is significantly negative in the short and long run. Therefore, an increase in current expenditure as a percent of GDP by one unit decreased the annual rate of change in GDP by (2.3055) unit in the short run and by (0.4620) unit in the long run even when other factors are held constant. This is because current expenditure in the economy represented by salaries and wages, subsidies, and transfers do not contribute to an increase in the productive capacity of the economy; thus, it is considered non-productive consumption expenditure. In the long run, current expenditures crowd out public and private investment; hence the growth rate will be very weak. It deprives nations from opportunities for realizing sustainable economic growth, particularly in the Iraqi economy, which heavily depends on oil revenues and does not have income diversification. Thus, an increase in current expenditures does not stimulate the economy in reality but rather it limits it from expanding.
This finding is consistent with the study by Al-Douri and Arhim (2016), ^4^ which concluded that the dominance of current spending in the Iraqi economy’s general budget does not contribute to economic growth because most of this spending is directed towards salaries and current transfers rather than investment spending.
2. The investment expenditure indicator as a percent of GDP (X2) indicates a positive and significant response between the investment expenditure indicator as a percent of GDP and the annual rate of change in GDP in both the short and long run. An increase in investment expenditure as a percent of GDP by one unit leads to an increase in the annual rate of change in GDP by (0.5942) units in the short run and by (13.44848) units in the long run, with other factors remaining constant. The result is consistent with the logic of economic theory. The model indicates that investment increases growth to a limited extent in the short run and its effect multiplies in the long run due to capital accumulation and increased productivity, which is consistent with Keynesian theory in stimulating aggregate demand and with the Solow model in enhancing productive capacity in the long run.
This finding aligns with Wulandari’s (2024) ^13^ study, which concluded that capital spending is a key driver of economic growth by improving infrastructure and increasing productive capacity.It also supports Al-Douri and Arhim’s (2016) ^4^ study, which emphasized the importance of directing available resources toward investment spending to achieve sustainable economic growth. This agreement suggests that restructuring public spending to favor investment is among the most important requirements for boosting growth in the Iraqi economy.
3. The education expenditure indicator as a percent of GDP (X3) shows a statistically significant positive effect at 1% between (X3) and economic growth annual rate of change in GDP in both the short and long run. Increasing the percent of education expenditure as a percent of GDP by one unit increases the annual rate of change in GDP by 22.60350 units in the short run and by 11.81809 units in the long run, other factors being constant. This is highly justified by economic logic that the acceleration of aggregate demand is the main stimulus of market growth and culture, and human capital is the most important source of national growth.
The results of the current study are consistent with the model of Mankiw, Romer, and Weil (1992), ^9^ which concluded that investment in human capital is one of the most important factors driving long-term economic growth. They also align with economic literature that recognizes that improving the quality of education leads to increased labor productivity and enhances the competitiveness of the economy. This underscores the importance of spending on education and its continuation as a productive investment, not merely a consumer expenditure.
4. The findings demonstrated a significant inverse correlation between health expenditure as a percent of GDP (X4) and economic growth (annual rate of change in GDP) in both the short and long run. This is consistent with the economic logic of the correlation between the two variables, as an increase in health expenditure as a percent of GDP by one unit leads to a decrease in the annual rate of change in GDP by (38.36932) units in the short run and by (70.22082) units in the long run. The increase in health expenditure in Iraq has often been a response to crises (wars, terrorism, epidemics) rather than productive investment expenditure. A large part goes to treatment abroad and imported medicines instead of building an effective domestic health system. Therefore, it demonstrates a negative impact on growth because it does not translate into real productivity.
This result differed from what is indicated by traditional economic literature, which acknowledges that health spending is an investment in human capital that contributes to increased productivity and economic growth. This difference can be explained by the unique characteristics of the Iraqi economy, as a large part of health spending during the study period is directed towards addressing the effects of crises and wars and financing operational expenses, rather than being directed towards productive health investments, which limited its positive impact on economic growth during the study period.
5. The findings demonstrated a significant direct correlation between the price of a barrel of oil (USD/barrel) (X5) and economic growth (annual rate of change in GDP) in the short run, and a non-significant inverse correlation in the long run. An increase in the price of a barrel of oil by one unit leads to an increase in the annual rate of change in GDP by (0.149584) units in the short run and a decrease in the annual rate of change in GDP by (0.057642) in the long run, with other factors remaining constant. Thus, in the short run, the direct correlation between oil prices and economic growth aligns with Keynesian theory, where higher oil revenues increased government spending and aggregate demand, directly impacting GDP. In the long run, the inverse (non-significant) correlation aligns with theories of Dutch Disease and the “resource curse” in economic thought, which argue that excessive reliance on oil weakens non-oil sectors and makes growth unsustainable, despite high oil revenues.
This finding aligns with the nature of the Iraqi economy as a rentier economy heavily reliant on oil revenues. While higher oil prices lead to increased government spending and public revenues, thus stimulating short-term economic growth, the weak long-term impact supports the resource curse hypothesis. This hypothesis suggests that excessive dependence on natural resources limits economic diversification and reduces the sustainability of economic growth.
6. The inflation rate index (X6) indicates a negative and significant effect of the inflation rate on the annual rate of change in GDP in both the short and long run, which is consistent with economic theory. An increase in the inflation rate by one unit leads to a decrease in the annual rate of change in GDP by (19.41467) units in the short run and by (0.347127) units in the long run, with other factors remaining constant, because high inflation reduces purchasing power and weakens economic stability, thus having a negative impact on economic growth.
The results of the current study are consistent with the study by Dahham and Maayouf (2022), ^10^ which concluded that imbalances in monetary stability negatively affect economic performance. They are also consistent with the economic theory that sees that high rates of economic inflation lead to the erosion of the purchasing power of money, an increase in uncertainty, and a decline in investment, which is reflected negatively on economic growth.

### Conclusions

a. The research hypothesis was proven through the findings of the cointegration test for the (ARDL) model, demonstrating a long-term equilibrium correlation between the independent variables and the dependent variable, economic growth, i.e., the existence of a cointegration correlation, where the F-statistic magnitude (F = 6.649300) was greater than its upper and lower critical magnitudes at a significance level of less than (1%).
b. The findings of the econometric analysis demonstrated a significant direct correlation between economic growth and (investment spending as a percent of GDP, education spending as a percent of GDP, price of a barrel of oil), which is consistent with the logic of economic theory regarding the correlation between economic variables.
c. The findings of the econometric analysis demonstrated a significant inverse correlation between economic growth and (current spending as a percent of GDP, health spending as a percent of GDP, inflation rate). This does not align with the logic of economic theory, except for the (inflation rate), which is consistent with the logic of economic theory regarding the correlation between the two variables.

### Recommendations

a. To diversify the economy and promote sustainable growth, increase investment spending to over 8% of GDP and prioritize productive sectors including agriculture, industry, and renewable energy.
b. Increase education spending to above 5% of GDP, prioritize quality, connect it to the labor market, and expand technical and vocational education programs to boost human capital and productivity for long-term economic growth.
c. To effectively manage oil earnings, diversify income sources, create a sovereign fund to invest surpluses, and adopt flexible fiscal policies to adapt to global price shocks.
d. Restructure expenditure by decreasing wasteful expenses, focusing on profitable sectors, and adopting rigorous governance and financial control mechanisms to improve public resource management efficiency and decrease waste and corruption.
e. To improve health spending efficiency, invest in infrastructure, adopt digital systems for resource management, increase transparency, and involve the private sector in financing and operating services to expand coverage and improve quality.
f. To maintain buying power stability, use conservative monetary policies to keep inflation within goal bounds (2%–3%), increase local production to reduce imported inflation, and link fiscal policies and wages to price levels.

## Ethical considerations

This study did not require ethical approval as it relied on secondary and publicly available data.

## Resources

## Data Availability

The data supporting the findings of this study are openly available in Zenodo at: [ https://doi.org/10.5281/zenodo.19666496], under the Creative Commons Attribution 4.0 International (CC BY 4.0) license. ^1^ Repository name: *Macroeconomic Indicators and Government Expenditure Structure in Iraq (2004–2023) *. [ https://doi.org/10.5281/zenodo.19666496]. ^1^ The project contains the following underlying data: Macroeconomic dataset for Iraq (2004–2023).xlsx (includes GDP growth rate, government expenditure components, oil prices, and inflation rates). Data are available under the terms of the Creative Commons Attribution 4.0 International license (CC BY 4.0). No extended data are associated with this study.
