# Supplementary material for: Analysis of the Impact of Disaggregates Government Expenditure on Economic Growth in Iraq
Source: F1000Res. 2026 Aug 21;15:1112. Originally published 2026 Jul 8. [Version 2] doi: 10.12688/f1000research.177997.2 (PMC13560066; doi:10.12688/f1000research.177997.2)
Supplement: Peer review history

# Reviewer report: Reviewer response for version 2

**DOI:** 10.5256/f1000research.207946.r513336

**Published:** 2026-08-25

**Authors:** Ibrahim Iliyasu

**Affiliations:** 1 Umaru Musa Yar’adua University Katsina State, Katsina State, Nigeria

the manuscript has substantially been improved following the revision made by the author(s)

Is the work clearly and accurately presented and does it cite the current literature?

Partly

If applicable, is the statistical analysis and its interpretation appropriate?

Yes

Are all the source data underlying the results available to ensure full reproducibility?

Yes

Is the study design appropriate and is the work technically sound?

Partly

Are the conclusions drawn adequately supported by the results?

Yes

Are sufficient details of methods and analysis provided to allow replication by others?

Partly

Reviewer Expertise:

public sector economics

I confirm that I have read this submission and believe that I have an appropriate level of expertise to confirm that it is of an acceptable scientific standard.

# Reviewer report: Reviewer response for version 1

**DOI:** 10.5256/f1000research.196328.r500471

**Published:** 2026-08-03

**Authors:** Ibrahim Iliyasu

**Affiliations:** 1 Umaru Musa Yar’adua University Katsina State, Katsina State, Nigeria

1. The paper studied the evolution of government current, investment, education and health expenditure in Iraq over the 2004-2023 periods. On the basis of descriptive statistics and bound test to cointegration analysis of quarterly data the author(s) reported mild increase in government expenditure components. In addition, the result confirmed long run equilibrium relationship among economic growth, expenditure components alongside inflation and oil price. Furthermore, government investment expenditure as share of GDP, education spending as share of GDP and oil price has an increasing effect on economic growth. While, current spending as share of GDP, health spending as share of GDP and inflation rate has a decreasing effect on economic growth. The paper concludes that there is a need to prioritised government investment spending over other expenditure component to accelerate the economic growth in Iraq.

 2. Since, the paper methodology did not take care of shocks and structural disturbance  among the variables, I suggest the paper tittle should be recast as **“Analysis of the Impact of Disaggregates Government Expenditure on Economic Growth in Iraq”**

 3.The introduction section should be improved upon for example, various sub-section and conceptual definitions are not necessary, since they are common terminologies. In addition the paper is not properly situated within the current states of knowledge on the topic and consequently the literature gap is not made explicit. Thus, authors need to provide a brief synthesis of the current state of knowledge on the topic and makes the literature gap more explicit.

 4. There is need to clearly separate the Methodology and Result and Discussion Sections. In addition the methodology section should be sequentially presented as follows (i) theoretical framework and model specification (ii) sources of data and variables description (iii) empirical strategy. Furthermore, author should replace X1 to X6 with appropriate abbreviation of the variables. Again there is possibility of structural breaks in the variable which authors failed to account in both unit root testing and ARDL modelling.

 5. In discussing the results, author(s) failed to compare the finding with previous studies. This is important as it will allow the paper contribution to the literature to be showcase. Further, author(s) are in love with sub-headings and numbering which make the paper presentation less coherent. It is advisable to minimize their usage.

 6. Also there is need to proofread the paper to improve flow and grammatical constructions

Is the work clearly and accurately presented and does it cite the current literature?

Partly

If applicable, is the statistical analysis and its interpretation appropriate?

Yes

Are all the source data underlying the results available to ensure full reproducibility?

Yes

Is the study design appropriate and is the work technically sound?

Partly

Are the conclusions drawn adequately supported by the results?

Yes

Are sufficient details of methods and analysis provided to allow replication by others?

Partly

Reviewer Expertise:

public sector economics

I confirm that I have read this submission and believe that I have an appropriate level of expertise to confirm that it is of an acceptable scientific standard, however I have significant reservations, as outlined above.

# Response

**Published:** 2026-08-03

**Authors:** مهند خميس عبد

((Reviewer's Comments))

 1. The first comment: The researchers thank the reviewer for this valuable observation, which points to the necessity of prioritizing government investment spending over other spending components to accelerate economic growth in Iraq. This has led to a 10.4% contraction in growth, reflecting the heavy reliance on oil revenues, which is inconsistent with the logic of economic theory during the research period (2004-2023). This is confirmed in the same paragraph of the research paper.

 2. The second comment: The research title has been changed to "A Detailed Analysis of the Impact of Government Spending on Economic Growth in Iraq."

 3. The third comment: Regarding improving the introduction section and addressing the literature gap, we believe we have done so to the best of our knowledge. Some reviewers consider this introduction necessary to provide a broad foundation for the theoretical concepts of the research variables under study.

 4. The fourth comment: Regarding presenting the methodology section in the following sequence: (a) Theoretical Framework and Model Definition, (b) Data Sources and Variable Description, (c) Experimental Strategy, the researchers have adhered to the journal's established publication methodology.

 5. The possibility of structural breaks in the variable was not considered by the researchers in either the unit root test or the ARDL model. This is because structural break tests were not used in this study, as the primary objective was to estimate the long-term relationship between variables using the ARDL model according to its standard methodology. This methodology does not necessarily require structural break tests unless there is clear statistical or theoretical evidence of a permanent change in the structure of the relationship between the variables under study.

 6. The researchers thank the reviewer for this valuable observation. The discussion of the results was based on their interpretation in light of theoretical frameworks and the Iraqi economic reality. However, the researchers agree on the importance of strengthening the discussion through direct comparison with the results of previous studies to highlight the scientific contribution of this study. These comparisons will be included in the revised version. As for the numerous subheadings and numbering, these were adopted to facilitate the presentation of the results and to distinguish the axes of analysis.

# Reviewer report: Reviewer response for version 1

**DOI:** 10.5256/f1000research.196328.r500470

**Published:** 2026-07-15

**Authors:** Junior Semuel Lakat

**Affiliations:** 1 Universitas Klabat, Manado, Indonesia

1. Limitations Regarding the Time Frame: The background section in abstract mentions the 2023–2024 crisis period, but the data used in the quantitative model only extends through 2023. The full-text article may need to explain how the dynamics of 2024 were analyzed (whether using projections or additional qualitative analysis).

 2. Anomalies in the Health Sector Warrant Further Exploration: The negative relationship between health spending and GDP is a highly intriguing yet counterintuitive finding. This article should provide additional qualitative explanations as to why this occurs in Iraq (for example: is it due to budgetary corruption, the post-war devastation of medical infrastructure, or bureaucratic inefficiencies?).

Is the work clearly and accurately presented and does it cite the current literature?

Yes

If applicable, is the statistical analysis and its interpretation appropriate?

Yes

Are all the source data underlying the results available to ensure full reproducibility?

Yes

Is the study design appropriate and is the work technically sound?

Yes

Are the conclusions drawn adequately supported by the results?

Yes

Are sufficient details of methods and analysis provided to allow replication by others?

Partly

Reviewer Expertise:

Economics, Public Financial Management.

I confirm that I have read this submission and believe that I have an appropriate level of expertise to confirm that it is of an acceptable scientific standard.

# Response

**Published:** 2026-08-03

**Authors:** مهند خميس عبد

((First Reviewer's Observations))

 1. The observation regarding the time period in the research was addressed; the error was typographical.

 2. The reviewer's second observation, which points to anomalies in the health sector, requires further qualitative explanation regarding why this occurred in Iraq. The researcher has already addressed this within the research in the following paragraph: Increases in health spending in Iraq have often been a response to crises (wars, terrorism, epidemics) rather than productive investment. A significant portion of this spending is allocated to treatment abroad and imported medicines instead of building an effective local health system. Therefore, this spending has a negative impact on growth because it does not translate into real productivity. This is documented within the research.
